# Country-specific reference values for PROMIS^®^ pain, physical function and participation measures compared to US reference values

**DOI:** 10.1080/07853890.2022.2149849

**Published:** 2022-11-25

**Authors:** Caroline B. Terwee, Leo D. Roorda

**Affiliations:** a Department of Epidemiology and Data Science, Vrije Universiteit Amsterdam, Amsterdam, The Netherlands; b Amsterdam Public Health Research Institute, Amsterdam, The Netherlands; c Amsterdam Rehabilitation Research Center | Reade, Amsterdam, The Netherlands

**Keywords:** Patient-reported outcomes, questionnaires, PROMIS, reference values, pain, physical function, participation

## Abstract

**Introduction:**

Patient-Reported Outcomes Measurement Information System (PROMIS^®^) is commonly used across medical conditions. To facilitate interpretation of scores across countries, we calculated Dutch reference values for PROMIS Physical Function (PROMIS-PF), Pain Interference (PROMIS-PI), Pain Behavior (PROMIS-PB), Ability to Participate in Social Roles and Activities (PROMIS-APSRA), and Satisfaction with Social Roles and Activities (PROMIS-SSRA), as compared to US reference values.

**Patients and methods:**

A panel completed full PROMIS-PF (*n*=1310), PROMIS-PI and PROMIS-PB (*n*=1052), and PROMIS-APSRA and PROMIS-SSRA (*n*=1002) item banks and reported their level of health per domain (no, mild, moderate, severe limitations). T-scores were calculated by sample and subgroups (age, gender, self-reported level of domain). Distribution-based and anchor-based thresholds for mild, moderate, and severe scores were determined.

**Results:**

Mean T-scores were close to the US mean of 50 for PROMIS-PF (49.8) and PROMIS-APSRA (50.6), lower for PROMIS-SSRA (47.5) and higher for PROMIS-PI (54.9) and PROMIS-PB (52.0). Distribution-based thresholds for mild, moderate, and severe scores were comparable to US recommended cut-off values (except for PROMIS-PI) but participants reported limitations ‘earlier’ than suggested thresholds.

**Conclusion:**

Dutch reference values were close to US reference values for some PROMIS domains but not all. We recommend country-specific reference values to facilitate worldwide PROMIS use.

**KEY MESSAGES**
- PROMIS offers universally applicable IRT-based efficient and patient-friendly measures to assess commonly relevant patient-reported outcomes across medical conditions.
- To support the use of PROMIS in daily clinical practice and research across the world, country-specific general population reference values should be obtained.
- More research is necessary to obtain reliable and valid cut-off values for what constitutes mild, moderate and severe scores from the patients’ perspective.

## Introduction

Patient-Reported Outcome Measures (PROMs) are increasingly used for outcome measurement in clinical practice to facilitate value-based health care. There is evidence that the routine use of PROMs can lead to better patient-clinician communication, increased discussion of psychosocial issues and improved shared-decision making [1–3]. In addition, beneficial effects of routine PROM use have been found on symptom control, quality of life outcomes, patient satisfaction and even survival [2,4–10] as well as on health care expenditure [11–13].

The beneficial effects of the routine use of PROMs can only be obtained if PROMs are successfully implemented in daily clinical care [14–16]. However, there are many implementation barriers. Important ones are the selection of patient-reported outcomes (PROs) that are most relevant for patients and the selection of the most suitable PROMs to measure these PROs. A quite common approach is to use disease-specific PROMs because it is assumed that these PROMs are most relevant for the patient group at issue and most responsive to their treatment. However, implementing disease-specific PROMs in daily clinical practice is doomed to fail. It is too time-consuming and too costly to implement disease-specific PROMS in electronic health records for every patient group. It is too complex for clinicians and patients to interpret and discuss PROMs with different scales and different cut-off values. And finally, it is too burdensome to ask an increasing number of patients with multiple conditions to complete multiple disease-specific PROMs, often with overlapping content.

Successful implementation of PROMs in routine clinical practice requires a shift towards measuring generic PROs with generic PROMs as much as possible, only supplemented with disease-specific PROMs for outcomes that are really disease-specific such as disease-specific symptoms. Two research findings show that such a shift is possible. First, it has been shown that PROs that matter most to patients are common across conditions [17–19]. Examples of commonly relevant outcomes are physical function, pain and participation. Second, it has been shown that generic PROMs developed within the modern framework of item response theory (IRT) [20,21], can have equal or even better responsiveness than traditional generic PROMs developed within the framework of classical test theory [22–28], especially when they are used as a computerized adaptive test (CAT), where the computer selects relevant questions based on answers to previous questions [29,30].

The Patient-Reported Outcomes Measurement Information System (PROMIS^®^) initiative has developed IRT-based PROMs to measure commonly relevant outcomes such as physical function, pain, fatigue, sleep disturbances, anxiety, depression and the ability to participate in social roles and activities. These PROMs are applicable to adults and children with or without (chronic) diseases [31–33]. PROMIS measures can be administered as fixed short forms or CAT. Evidence for sufficient psychometric properties across patient populations is growing [34–40]. PROMIS measures have been translated into more than 60 languages and are increasingly used across countries [41]. For example, Dutch-Flemish translations of PROMIS measures are available for more than 30 domains and have been validated in different populations [42–52]. PROMIS has recently been recommended as the preferred measurement system for assessing commonly relevant PROs in Dutch daily medical specialty care across patient conditions [53].

To support the use of PROMIS in daily clinical practice and research, reference values from the general population are useful. Most PROMIS measures were centered to have a mean of 50 and an SD of 10 in the US general population. However, the health of populations may be different in other countries so it is useful to assess to what extent references values are similar across countries. Therefore, we aimed to obtain general population-based Dutch reference values for five PROMIS domains: Physical Function, Pain Interference, Pain Behavior, Ability to Participate in Social Roles and Activities and Satisfaction with Social Roles and Activities and compare them with US reference values.

## Patients and methods

### Study participants

A data collection company (Desan Research Solutions) recruited three waves of at least 1000 people from the Dutch general population from an existing internet panel in 2016. The panel was provided by Global Market Insite (GMI). Informed consent to become a panelist was obtained by GMI. Panelists were recruited by an invitation from the panel host to participate. By voluntarily responding to the invitation for this survey, panelists provided informed consent to participate in the study. More details about the panel are provided by Elsman et al. [54]. The study samples were selected to be representative of the Dutch general population with respect to age distribution (18–40; 40–65; >65), gender, educational level (low, middle, high), region of residence (north, east, south, west) and ethnicity (native Dutch, first- and second-generation western immigrant, first- and second-generation non-western immigrant).

### Procedures

A web-based survey was used, in which skipping items was not allowed. Participants were asked to complete an online questionnaire once. In Wave 1 participants completed the full v1.2 PROMIS Physical Function item bank, in Wave 2 participants completed the full v1.1 PROMIS Pain Interference and v1.1 Pain Behavior item banks, and in Wave 3 participants completed the full v2.0 PROMIS Ability to Participate in Social Roles and Activities and Satisfaction with Social Roles and Activities item banks. Additionally, participants were asked to describe their level of health for each domain on a single item, described below. Afterwards, participants completed questions regarding sociodemographic characteristics (age, gender, education, region of residence and ethnicity). The Medical Ethical Committee of Amsterdam UMC, location VUmc, the Netherlands, confirmed that the study protocol was exempted from ethical approval according to the Dutch Medical Research in Human Subjects Act (WMO), as no experiments were conducted.

### Measures

The PROMIS v1.2 Physical Function item bank contains 121 items, measuring the ability to perform activities including upper extremities (dexterity), lower extremities (walking or mobility) and central regions (neck, back), as well as the ability to perform instrumental activities of daily living, such as running errands. The PROMIS v1.1 Pain Interference item bank contains 40 items referring to the self-reported consequences of pain on relevant aspects of one’s life, including the extent to which pain hinders engagement with social, cognitive, emotional, physical and recreational activities. The PROMIS v1.1 Pain Behavior item bank contains 39 items referring to verbal or non-verbal and involuntary or deliberate self-reported external manifestations of pain: behaviors that typically indicate to others that an individual is experiencing pain. The PROMIS v2.0 Ability to Participate in Social Roles and Activities item bank contains 35 items measuring the perceived ability to perform one’s usual social roles and activities. The PROMIS v2.0 Satisfaction with Social Roles and Activities item bank contains 44 items measuring satisfaction with performing one’s usual social roles and activities. In the Physical Function, Pain Interference and both Participation item banks five response options are used. In the Pain Behavior item bank six response options are used (including the option ‘had no pain’). The Physical Function item bank and both Participation items banks have no time frame. The Pain item banks use the past 7 days as a time frame. All item banks are scored on a T-score metric, which has an average of 50 and standard deviation (SD) of 10 in the US general population. Higher scores indicate more of the construct being assessed. For example, higher Physical Function scores indicate better physical function, demonstrating good health, whereas higher Pain Interference scores indicate more pain interference, representing poor health.

Five single items were used to measure the overall level of the health domains, one item for each domain (physical function, pain interference, pain behavior, ability to participate in social roles and activities and satisfaction with social roles and activities). For example: ‘How would you describe your physical function?’. Response options for all five items were: no limitations, mild limitations, moderate limitations and severe limitations.

### Statistical analyses

First, we compared the characteristics of the study participants to data from Statistics Netherlands in 2016 [55] to check for a maximum allowable deviation of 2.5% per sociodemographic variable. Second, we compared our data to a US general population sample to ensure that T-scores of comparable Dutch and US populations can be compared unbiasedly. We used PROMIS wave 1 data, obtained from the HealthMeasures Dataverse repository [56]. We only selected people from the general population (Physical Function *n* = 1700, Pain Interference *n* = 946, Pain Behavior *n* = 881, Ability to Participate in Social Roles and Activities *n* = 429, Satisfaction with Social Roles and Activities *n* = 424). In this DIF analysis, we examined whether Dutch and US people with the same level of domain have different probabilities of giving a certain response to an item [57]. We performed Differential Item Functioning (DIF) analyses by comparing a series of ordinal logistic regression models, using the R package Lordif (version 0.3-3) [58]. We used McFadden’s pseudo *R*^2^ change of 2% between the models as a criterion for DIF. Uniform DIF exists when the magnitude of the DIF is consistent across the entire range of the trait. Non-uniform DIF exists when the magnitude or direction of DIF differs across the trait. We checked the impact of DIF on total scores by examining test characteristic curves, displaying the difference between the groups when calculating a total raw score based on all items or on items flagged for DIF only.

Third, we calculated PROMIS T-scores per item bank from the raw item scores using the online HealthMeasures Scoring Service program, provided by the US Assessment Center [59]. All participants, including people who reported ‘had no pain’ on the Pain Behavior item bank were included in the analyses. T-scores were calculated for the entire sample, as well as for subgroups based on age (18–34 years, 35–44 years, 45–54 years, 55–64 years, 65–74 years and ≥75 years), gender and self-reported level of the domain (anchor-based thresholds). We also calculated distribution-based thresholds for mild, moderate and severe T-scores based on 0.5 × SD, 1 × SD and 2 × SD below (for constructs indicating good health) or above (for constructs indicating poor health) the average of the general population, respectively. We compared the mean T-scores of the Dutch and US populations and the subgroups. For the Physical Function and Pain item banks, we used gender and age range sub-norms for adult PROMIS measures centered on the US General Census 2000, presented on the HealthMeasures website [60]. For the Participation item banks, we calculated T-scores using the US PROMIS 1 Social Supplement, obtained from the HealthMeasures Dataverse repository [56]. We selected only the participants from this Supplement who were recruited from the US general population (Polimetrix sample, *n* = 1008).

## Results

### Study participants

The three waves included 1310 (Physical Function), 1052 (Pain) and 1002 (Participation) participants, respectively. Characteristics of the participants are summarized and compared to the Dutch population in 2016 in Table 1. All differences were less than the 2.5% agreed upon.

**Table 1.** Sociodemographic characteristics of study participants and the Dutch general population.

Sociodemographic characteristics^a^	Study participants Wave 1 Physical function (*n* = 1310)	Study participants Wave 2 Pain interference & pain behavior (*n* = 1052)	Study participants Wave 3 Ability to participate & satisfaction with participation (*n* = 1002)	Dutch adult population 2016^b^(*n* = 13.6 million)
Age in years, mean ± SD (range)	51 ± 17 (19–87)	51 ± 17 (19–87)	51 ± 17 (19–89)	
18–39	35	32	31	34
40–65	42	45	46	44
>65	23	23	23	23
Gender				
Male	47	47	48	49
Female	53	53	52	51
Educational level				
Low	31	28	30	30
Middle	40	40	40	40
High	29	32	30	30
Region of residence				
North	9	10	10	10
East	21	22	23	21
South	21	21	22	22
West	49	47	47	47
Ethnicity				
Native	79	77	79	79
1st and 2nd generation western immigrant	12	12	10	10
1st and 2nd generation non-western immigrant	9	11	11	11

^a^All results are expressed as percentages (%) unless otherwise noted.

^b^Based on data from Statistics Netherlands (https://www.cbs.nl).

SD: standard deviation.

### Comparability of Dutch and US scores

Two items of the Physical Function item bank and two items of the Pain Behavior item bank were flagged for uniform DIF (Table 2). In both cases, for one item the Dutch population endorses higher item response categories at the same level of the domain than the US population, and for the other item, it was the other way round. The impact of DIF on the total scores was considered negligible. No DIF was found for the other item banks.

**Table 2.** Items with DIF and their McFadden’s pseudo *R*^2^ and IRT parameters.

Item bank	Item with DIF	McFadden’s pseudo *R*^2^	Slope; threshold parameters^a^	Included in CAT simulation	Included in SF
Physical function	PFB5r1: Does your health now limit you in hiking a couple of miles (3 km) on uneven surfaces, including hills?	*R*^2^_12_ = 0.0208	**NL**: 3.29; −1.56, −1.02, −0.57, −0.12	45%	
		*R*^2^_23_ = 0.0009	US: 4.04; −1.12, −0.77, −0.26, 0.22		
	PFC29: Are you able to walk up and down two steps?	*R*^2^_12_ = 0.0341	NL: 2.29; −2.01, −1.74, −1.06	0%	
		*R*^2^_23_ = 0.0023	**US**: 3.08; −2.57, −2.08, −1.57		
Pain behavior	PAINBE50: When I was in pain I moved my limbs protectively	*R*^2^_12_ = 0.0236	NL: 3.64; −0.73, 0.59, 0.99, 1.54, 2.15	0%	
		*R*^2^_23_ = 0.0012	**US**: 3.81; −0.83, 0.28, 0.60, 1.17, 1.63		
	PAINBE26: Pain caused me to curl up in a ball	*R*^2^_12_ = 0.0356	**NL**: 4.72; −0.69, 0.52, 0.90, 1.47	0.002%	
		*R*^2^_23_ = 0.0048	US: 4.72; −0.74, 0.95, 1.31, 1.83		

^a^The bold population had lower thresholds compared to the other population, indicating that this population endorses higher item response categories at the same level of the domain.

CAT: Computerized Adaptive Test; SF: short form; NL: Netherlands; US: Unites States.

### Dutch PROMIS reference scores

Mean T-scores for the entire samples, and age and gender groups, for the five-item banks are presented in Tables 3 through 5. Mean T-scores in the Dutch general population were close to the mean T-scores in the US population of 50 for Physical Function (49.8) and Ability to Participate in Social Roles and Activities (50.6). However, the Dutch population showed lower levels of Satisfaction with Social Roles and Activities (47.5) and higher levels of Pain Interference (54.9) and Pain Behavior (52.0) than the US population.

**Table 3.** PROMIS Physical Function Dutch reference values by age and gender and compared with the US reference population [61].

	Dutch population, *n* (%)	US population, *n* (%)	Dutch mean T-score (SD)^a^	US mean T-score (SD)
Total	1310 (100)	3407 (100)	49.8 (10.8)	50.0 (10.0)
Gender				
Male	691 (47)	1363 (40)	50.9 (11.2)	51.7 (9.7)
Female	689 (53)	2044 (60)	48.8 (10.3)	48.9 (10.0)
Age in years				
18–34	282 (22)	782 (23)	55.2 (9.5)	55.1 (8.4)
35–44	214 (16)	605 (18)	52.8 (10.5)	52.0 (9.8)
45–54	199 (15)	567 (17)	50.0 (11.5)	49.0 (10.4)
55–64	279 (21)	565 (16)	46.4 (10.1)	47.5 (10.4)
65–74	280 (22)	457 (13)	46.7 (9.6)	47.2 (9.0)
75+	56 (4)	431 (13)	42.6 (9.5)	45.6 (8.5)
Distribution-based thresholds (based on SD)				
Within normal limits	885 (68)		>45	>45
Mild	174 (13)		39–45	40–45
Moderate	217 (17)		28–39	30–40
Severe	34 (2)		<28	<30
Anchor-based thresholds (based on self-reported limitations)				
No limitations	527 (40)		59.0 (7.0)	
Mild	452 (35)		48.1 (5.6)	
Moderate	259 (20)		39.4 (5.7)	
Severe	72 (5)		30.1 (6.6)	

^a^Higher scores represent a better physical function.

SD: standard deviation.

**Table 4.** PROMIS Pain Interference and Pain Behavior Dutch reference values by age and gender and compared with the US reference population [61].

	Pain interference				Pain behavior			
	Dutch population, *n* (%)	US population, *n* (%)	Dutch mean T-score (SD)^a^	US mean T-score (SD)^a^	Dutch population, *n* (%)	US population, *n* (%)	Dutch mean T-score (SD)^a^	US mean T-score (SD)^a^
Total	1052 (100)	3036 (100)	54.9 (8.6)	50.0 (10.0)	1052 (100)	3050 (100)	52.0 (9.4)	50.0 (10.0)
Gender								
Male	499 (47)	1180 (39)	54.7 (8.7)	48.3 (9.3)	499 (47)	1199 (39)	51.7 (9.5)	49.0 (9.7)
Female	553 (53)	1856 (61)	55.0 (8.5)	51.1 (10.3)	553 (53)	1851 (61)	52.3 (9.4)	50.7 (10.1)
Age in years								
18–34	200 (19)	712 (23)	52.3 (8.9)	47.8 (9.0)	200 (19)	699 (23)	49.5 (11.0)	47.6 (10.2)
35–44	174 (16)	548 (18)	54.5 (8.7)	50.1 (10.2)	174 (16)	561 (18)	52.7 (9.1)	50.0 (10.6)
45–54	156 (15)	499 (17)	55.9 (8.2)	51.9 (11.1)	156 (15)	507 (17)	53.3 (9.0)	52.2 (10.1)
55–64	239 (23)	488 (16)	56.6 (8.4)	51.6 (10.9)	239 (23)	507 (17)	53.1 (9.1)	51.3 (9.7)
65–74	223 (21)	406 (13)	55.0 (8.2)	49.9 (9.3)	223 (21)	402 (13)	52.1 (8.5)	50.1 (9.3)
75+	60 (6)	383 (13)	54.1 (8.1)	49.7 (8.7)	60 (6)	374 (12)	50.9 (8.8)	49.7 (8.7)
Distribution-based thresholds (based on SD)								
Within normal limits	695 (66)		<59	<55		641 (61)	<57	<55
Mild	203 (19)		59–63	55–60	322 (31)	57–61	55–60	
Moderate	148 (14)		63–72	61–70	85 (8)	61–71	61–70	
Severe	8 (1)		>72	>70	4 (0)	>71	>70	
Anchor-based thresholds (based on self-reported limitations)								
No limitations	238 (23)		45.9 (6.8)		168 (16)		44.0 (10.8)	
Mild	456 (43)		54.1 (6.0)		548 (52)		51.4 (8.0)	
Moderate	264 (25)		60.2 (5.6)		277 (26)		56.2 (7.7)	
Severe	94 (9)		66.2 (5.1)		59 (6)		61.4 (4.6)	

^a^Higher scores represent more pain interference/pain behavior.

SD: standard deviation.

**Table 5.** PROMIS Ability to Participate in Social Roles and Activities and Satisfaction with Social Roles and Activities Dutch reference values by age and gender and compared with the US reference population [61].

	Ability to Participate in Social Roles and Activities				Satisfaction with Social Roles and Activities			
	Dutch population, *n* (%)	US population, *n* (%)	Dutch mean T-score (SD)^a^	US mean T-score (SD)^a^	Dutch population, *n* (%)	US population, *n* (%)	Dutch mean T-score (SD)^a^	US mean T-score (SD)^a^
Total	1002 (100)	940 (100)	50.6 (9.5)	50.0 (9.8)	1002 (100)	922 (100)	47.5 (8.3)	50.0 (9.8)
Gender								
Male	477 (48)	335 (36)^b^	51.2 (9.5)	49.6 (9.2)	477 (48)	336 (36)^d^	48.1 (8.1)	50.2 (9.2)
Female	525 (52)	487 (52)^b^	50.1 (9.5)	49.8 (10.1)	525 (52)	487 (53)^b^	46.9 (8.4)	50.0 (10.2)
Age in years								
18–34	217 (22)	91 (10)^c^	51.5 (9.5)	49.7 (9.3)	217 (22)	92 (10)^d^	48.6 (8.1)	50.8 (9.1)
35–44	136 (13)	76 (8)^c^	48.4 (9.6)	51.6 (8.3)	136 (13)	76 (8)^c^	45.0 (8.4)	50.2 (9.0)
45–54	171 (17)	145 (15)^c^	51.1 (9.4)	47.5 (10.8)	171 (17)	145 (16)^c^	46.6 (8.3)	48.4 (10.3)
55–64	208 (21)	230 (24)^c^	49.4 (9.9)	49.2 (9.8)	208 (21)	230 (25)^c^	46.9 (8.5)	49.4 (10.0)
65–74	217 (22)	210 (22)^c^	51.4 (8.4)	51.0 (9.8)	217 (22)	210 (23)^c^	48.7 (7.4)	51.9 (10.0)
75+	53 (5)	69 (7)^c^	52.5 (11.0)	50.0 (8.0)	53 (5)	69 (7)^c^	49.6 (9.3)	49.6 (8.1)
Distribution-based thresholds (based on SD)								
Within normal limits	682 (68)		>46	>45	726 (73)		>43	>45
Mild	185 (19)		41–46	40–45	115 (11)		39–43	40–45
Moderate	114 (11)		32–41	30–39	119 (12)		31–39	30–39
Severe	21 (2)		<32	<30	42 (4)		<31	<30
Anchor-based thresholds (based on self-reported limitations)								
No limitations	430 (43)		56.8 (7.7)		430 (43)		51.9 (7.2)	
Mild	319 (32)		49.3 (6.4)		337 (34)		47.4 (5.0)	
Moderate	184 (18)		44.1 (6.7)		168 (17)		41.6 (5.8)	
Severe	69 (7)		35.7 (7.1)		67 (7)		34.0 (9.2)	

^a^Higher scores represent more ability to participate/satisfaction with participation; ^b^12% missing values; ^c^14% missing values; ^d^11% missing values.

SD: standard deviation.

Men had slightly better Physical Function and Participation scores than women (about 2 T-score points and 1 T-score point, respectively), while differences in Pain between men and women were less than 1 point. Physical Function levels were worst in the highest age groups, while Pain and Participation levels were worst in the middle age groups (45–64 years).

**Figure 1.**
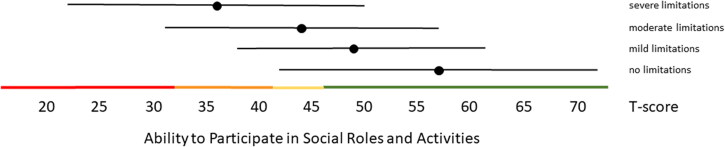
Mean Physical Function T-scores (±1.96 × SD) for people with self-reported no, mild, moderate and severe limitations. Colored lines indicate the current recommended Dutch PROMIS distribution-based thresholds (green = within normal limits, yellow = mild, orange = moderate, red = severe functional limitations).

**Figure 2.**
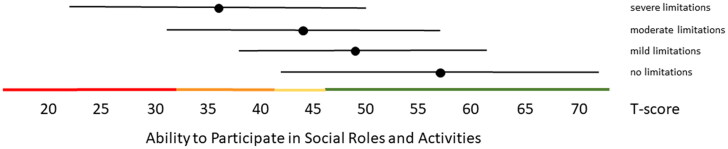
Mean Ability to Participate in Social Roles and Activities T-scores (±1.96 × SD) for people with self-reported no, mild, moderate and severe limitations. Colored lines indicate the current recommended Dutch PROMIS distribution-based thresholds (green = within normal limits, yellow = mild, orange = moderate, red = severe limitations in participation).

**Figure 3.**
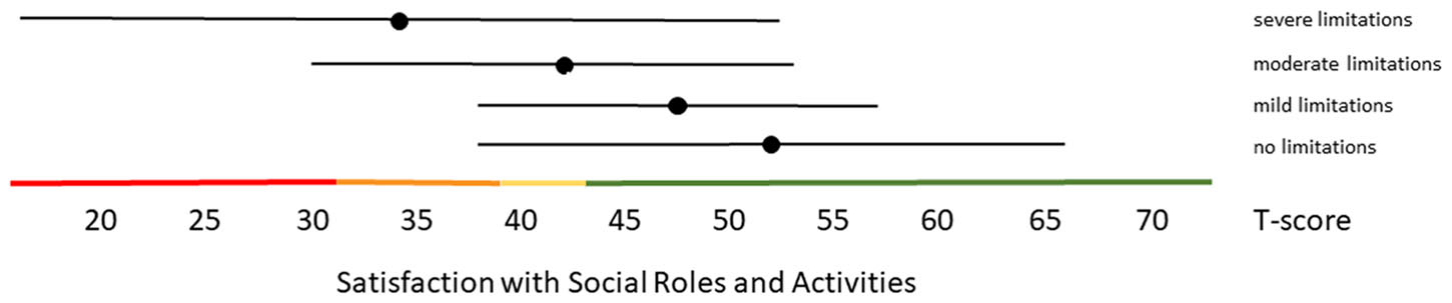
Mean Satisfaction with Participation in Social Roles and Activities T-scores (±1.96 × SD) for people with self-reported no, mild, moderate and severe limitations. Colored lines indicate the current recommended Dutch PROMIS distribution-based thresholds (green = within normal limits, yellow = mild, orange = moderate, red = severe limitations in participation).

**Figure 4.**
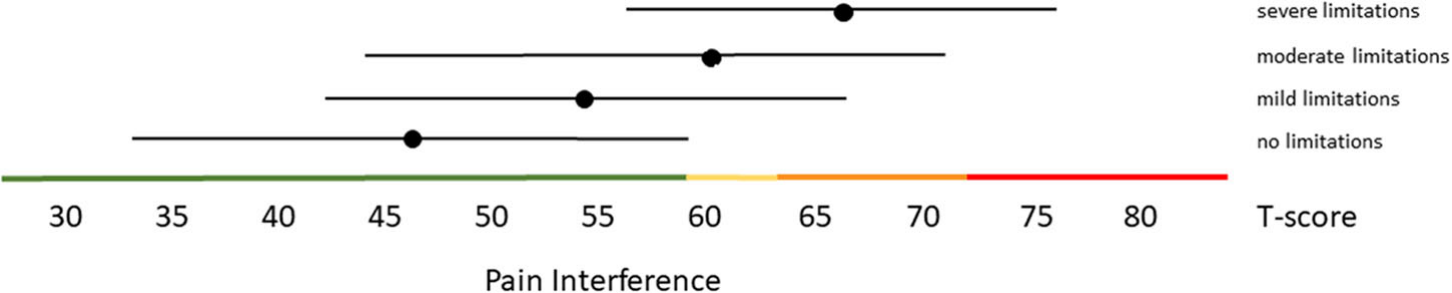
Mean Pain Interference T-scores (±1.96 × SD) for people with self-reported no, mild, moderate and severe limitations. Colored lines indicate the current recommended Dutch PROMIS distribution-based thresholds (green = within normal limits, yellow = mild, orange = moderate, red = severe symptoms).

**Figure 5.**
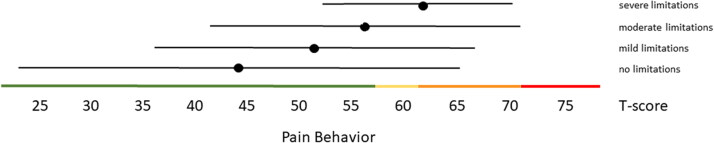
Mean Pain Behavior T-scores (±1.96 × SD) for people with self-reported no, mild, moderate and severe limitations. Colored lines indicate the current recommended Dutch PROMIS distribution-based thresholds (green = within normal limits, yellow = mild, orange = moderate, red = severe symptoms).

Distribution-based thresholds for mild, moderate and severe scores based on 0.5 × SD, 1.0 × SD and 2.0 × SD below (for constructs indicating good health) or above (for constructs indicating poor health) the average of the general population were found to be quite similar in the Dutch population as the suggested thresholds for the US population on the HealthMeasures website for Physical Function, Pain Behavior and both Participation item banks (Tables 3–5). For Pain Interference the thresholds were a bit higher in the Dutch population compared to the recommended US values because of the higher mean Pain Interference T-score in the Dutch population. However, anchor-based thresholds, based on mean T-scores for people who self-reported mild, moderate and severe limitations did not coincide with the distribution-based thresholds (Figures 1–5). Overall, people reported limitations ‘earlier’ (at lower severity levels) than the distribution-based cut-off values. For example, the mean T-scores for people who reported having mild symptoms/functional problems would be classified as within normal limits based on SD cut-off values for all domains, mean T-scores for people who reported having moderate symptoms/functional problems would be classified as mild problems based on SD cut-off values, and mean T-scores for people who reported to have severe symptoms/functional problems would be classified as moderate problems based on SD cut-off values. However, there was wide variation in T-scores within each self-reported limitations subgroup and there was wide overlap in T-score ranges between the subgroups.

## Discussion

This study assessed to what extent general population reference values for interpreting PROMIS T-scores were similar in the Netherlands as in the US. Mean T-scores in the Dutch general population were found to be close to the mean T-scores in the US population of 50 for Physical Function (49.8) and Ability to Participate in Social Roles and Activities (50.6). However, the average T-scores in the Dutch population were lower for Satisfaction with Social Roles and Activities (47.5) and higher for Pain Interference (54.9) and Pain Behavior (52.0). Distribution-based thresholds for mild, moderate and severe scores were comparable to the US recommended cut-off values for most item banks (except Pain Interference) but study participants reported limitations ‘earlier’ than these suggested distribution-based thresholds.

Only two items of the Physical Function item bank and two items of the Pain Behavior item bank were flagged for DIF, and the impact of DIF on T-scores was considered negligible, indicating that T-scores of comparable Dutch and US populations can be compared unbiasedly. These results are consistent with previous studies in clinical populations [40,44,45,62,63].

Two other studies reported mean T-scores in general population samples from the UK, France, Germany and Norway [64,65]. In the UK, France and Germany slightly higher mean T-scores (about 51–53) were found for Physical Function as compared to the Netherlands (mean T-score 49.8) and lower mean T-scores were found for Pain Interference (about 49–51) as compared to the Netherlands (mean T-score 54.9). The study from Norway also reported a mean T-score of 55.0 for Pain Interference, but a lower score for the Ability to Participate (48.3) as compared to the Netherlands (50.6). However, the Norwegian sample was not representative of the Norwegian general population. These studies and our study suggest that it is useful to obtain country-specific reference values for using PROMIS across countries. However, variables, other than country, could also be responsible for the differences in T-scores found between countries. For example, the US values are based on data collected in 2000, and the (perception of) population health may have changed over time. An alternative to country-specific reference values could be to base reference values on a multi-national data set. However, it is questionable whether this is achievable and, more importantly, whether a ‘world average’ would be meaningful.

The self-reported limitations by the study participants suggest that thresholds based on SDs may not be a valid indicator of what patients consider mild, moderate, or severe problems. Anchor-based thresholds based on patients’ opinions are generally considered more valid that distribution-based thresholds [66]. However, the self-reported limitations in this study were based on a single item only and given the wide variation in T-scores within each self-reported limitations subgroup and the wide overlap in T-score ranges between the subgroups, the validity of the self-reported limitations could be questioned. Previous studies have used a qualitative bookmarking methodology, which includes a ranking of clinical vignettes (i.e. descriptions of health states based on a selection of item responses) by patients or clinicians [67]. Using this method, Bingham et al. found thresholds for Pain Interference of 52, 63 and 72 for mild, moderate and severe Pain Interference, respectively, in RA patients [68]. Cella et al. found comparable thresholds of 50 for mild, 60 for moderate and 70 for severe Pain Interference in oncology patients [69]. We found no studies using this method on the other item banks included in this study. More research is necessary to obtain reliable and valid cut-off values for what constitutes mild, moderate and severe scores from the patients’ perspective. For the time being, we recommend using the distribution-based thresholds, consistent with the HealthMeasures recommendations. However, since our data are representative of the Dutch general population, we recommend using the Dutch distribution-based thresholds, obtained in this study, in the Netherlands, unless or until there is sound evidence that this is inappropriate. However, clinicians and researchers should keep in mind that less severe scores may also be considered problematic by patients.

The PROMIS domains addressed in this study are part of the eight PROMIS profile domains, which are considered the most important outcomes across (clinical) populations [70]. Dutch reference scores for the additional PROMIS profile domains Fatigue, Anxiety and Depression, as well as for the PROMIS Global Health Scale are published elsewhere or submitted for publication [54,71,72] and analyses of Dutch reference scores for the Sleep item banks are ongoing.

A strength of this study was the use of large and representative study samples. As indicated above, a limitation of this study was the use of only single items to measure self-reported limitations. Another limitation was that the maximum allowable deviation of 2.5% per sociodemographic variable for comparing the characteristics of the study participants to data from Statistics Netherlands was chosen arbitrarily. We could not find any recommendations for an acceptable deviation from a reference population in the literature. Furthermore, the study was only performed in the Netherlands, while the PROMIS measures are also used in Flanders, the Dutch-speaking part of Belgium. One study investigated DIF for the two pain item banks between Dutch and Flemish RA patients, and found only one item with DIF, with negligible impact [73]. Therefore the reference values obtained in our study may also be relevant for the Flemish population. However, future studies may be needed to investigate whether population levels of pain, function and participation are similar in the Netherlands and Flanders. A final potential limitation of the study was that the data was collected in 2016 and before the COVID-19 pandemic. Current population levels of pain, function and participation may be different. Ideally, reference values should be updated periodically (for example, the Public Health Monitor 2020 of the Dutch Community Health Services, Statistics Netherlands and the National Institute for Public Health and the Environment is updated every four years), but this is dependent upon funding.

## Conclusion

This study showed that general population reference values for interpreting PROMIS T-scores were close to US reference values for some PROMIS domains but not all. We recommend obtaining country-specific reference values for using PROMIS across the world. We also recommend using Dutch distribution-based thresholds for mild, moderate and severe scores, but keep in mind that less severe scores may also be considered problematic by patients. More studies are needed to define thresholds based on patients’ opinions.

## Data Availability

The dataset is available upon request from the corresponding author.
